# Outcome of Treatment of Osteoarthritis with Arthroscopic Debridement and Autologous Conditioned Plasma

**DOI:** 10.5704/MOJ.1703.008

**Published:** 2017-03

**Authors:** CKK King, A Yung

**Affiliations:** Department of Orthopaedics, Changi General Hospital, Singapore

**Keywords:** osteoarthritis, autologous conditioned plasma, arthroscopy, debridement

## Abstract

**Introduction:**

Worldwide estimates are that 9.6% of men and 18.0% of women aged over 60 years have symptomatic osteoarthritis. The current treatment options vary from conservative to joint replacement. Recently, debridement of the joint has become an option for symptomatic relief. We evaluated the outcome of arthroscopic debridement with autologous conditioned plasma. The latter helps to promote cellular repair. We have evaluated our results over a two year period.

**Materials and Methods:**

We retrospectively analyzed a cohort of 52 patients who underwent arthroscopic knee debridement with autologous conditioned plasma in 2011. The patients were followed up in clinic till discharge. The case notes were reviewed and baseline demographic data obtained. This included age, medical history, occupation, range of movement, BMI measurements, duration of operation and radiographic scores. We analyzed the outcomes against those factors.

**Results:**

Of the 52 patients in our study, 16 were female and 36 were male. The mean follow-up period in the clinic was 6.5 months. The Kellgren-Lawrence score was 21.2% Grade 1, 13.5% Grade 2, 51.9% Grade 3 and 13.5% Grade 4. Improvement in range of movement was seen in 32.7% of patients.

**Conclusions:**

This study shows that arthroscopic debridement with autologous conditioned plasma (ACP) has a role to play in the treatment of osteoarthritis. In view of these findings, we recommend that surgeons should consider arthroscopic debridement with autologous conditioned plasma as part of their treatment armamentarium.

## Introduction

The World Health Organization defines osteoarthritis as a degenerative joint disease which mainly affects the articular cartilage^1^. Osteoarthritis (OA) is a leading cause of disability and impaired quality of life in developed countries^2,3^. Moreover, it is expected that the prevalence of OA will increase by 7% in the proportion of adults above 65 years of age by 2030 in the United States, raising the total to 20% of the population^4^. In the United States, symptomatic knee occurs in 12% of adults age 60 years or older and in 6% of all adults age 30 years or older^5^. Classic findings of OA on plain radiographs are osteophytes, joint-space narrowing, subchondral sclerosis, and in more advanced disease, bone cysts.

Several risk factors have been identified for OA. These factors either cause excessive forces to be applied across the joint or cause disruption to the protective mechanisms of the joint, making them dysfunctional. Systemic risk factors would include advancing age, gender and genetic configuration, while local factors would include anatomy, trauma, body mass index, repetitive use and bone density.

Treatment options for osteoarthritis have been classified into conservative or surgical. The non-surgical treatment would be pain relief and improvement of physical functions. Acetaminophen and nonsteroidal anti-inflammatory drugs are the most commonly used agents. For patients with intermittent and mild symptoms, a combination approach to treatment with physical therapy and occupational therapy will help. Other options would include procedures such as intra-articular injections of corticosteroid or hyaluronic acid. However, these should not be repeated more than a few times because of association with an increased risk of cartilage breakdown^6,7^. Surgical options would include joint arthroscopy and joint arthroplasty.

The objective of this study was to assess the benefit of arthroscopic debridement with autologous conditioned plasma on osteoarthritic knee as a treatment option.

## Materials and Methods

All patients who underwent arthroscopic knee surgeries by the same surgeon over a one year period between January 2011 to December 2011 were reviewed. Patients were excluded from the study if they have had other procedures and treatments such as anterior cruciate reconstruction, metabolic bone disease, fractures and inflammatory, infection, or neuropathic arthropathy. The radiographs of the symptomatic knee were rated using the Kellgren-Lawrence scale^8^. All subjects had weight-bearing knee radiographs.

Data were retrospectively collected from patient records on parameters that included age, occupation, mechanism of injury, past medical history, use of steroids, length of time off work, period with pain and time to presentation for consultation, side of surgery and duration of operation.

The same surgeon performed all the surgical procedures. The patient would have an arthroscopic examination, lavage, debridement and some would also have meniscal repairs followed by autologous conditioned plasma injection. The system for autologous conditioned plasma (ACP) injection was similar for all patients. Arthrex ACP double syringe system was used. ACP would increase the concentration of platelets and growth factors which are associated with the healing process.The blood from the patient would be withdrawn before the start of the surgery, followed by centrifugation and the ACP would be injected at the end of the surgery. The post-operative rehabilitation protocol for arthroscopic debridement with ACP would be for weight bearing as tolerated immediately after surgery. The initial exercises for the first two weeks would be straight leg raise, quadriceps exercise and knee flexion and extension exercises. After two weeks, the patients would be progressed to walking and training in the gym. Patients who had meniscal repair, would be non-weight bearing for six weeks, but allowed the initial exercise regime. Following the six weeks, they would progress to full weight bearing with walking and training exercises.

Patients were retrospectively followed up from the time of entry in the case records at first visit till the time of discharge from clinic. The case records were traced to see if the patient presented with similar symptoms after discharge or had another surgery after the index surgery on their knee. The study participants were assessed for their range of motion at the end of their follow up.

The primary clinical outcome measure was the patients’ symptoms of pain in their knee and their range of motion, and if there was any progression of their symptoms at discharge. Failure was defined as either recurrence of pain or need for reoperation.

The data was analyzed using IBM SPSS Statistics™ Version 20 (IBM Corp, Armonk). Categorical variables are presented as percentages and compared using Pearson’s chi-squared test or Fisher’s exact test where appropriate. Continuous variables are presented using the mean (± standard deviation), and comparisons were performed by utilizing the unpaired t-test. The null hypothesis was that there was no association between the tested variable and symptom progression. Symptom progression was defined as a worsening pain or undergoing another surgery on the affected knee. Statistical significance was defined as p<0.05. Baseline characteristics were compared between those that had their symptoms improved and those that presented with worsening or similar previous symptoms. Kaplan-Meier analysis was done on the data collected. It was assumed that patient who did not present again in the clinic with symptoms after discharge from clinic to have had a follow up till the point of June 2013. Further analysis was done with the Kellgren-Lawrence radiographic grading scores and the outcome measures. The radiographic grading scores were dichotomized into two groups, namely one with minimal OA changes (Kellgren-Lawrence scores of 0-2) and the other with more advanced osteoarthritis changes (Kellgren-Lawrence scores of 3-4).

## Results

The study participants comprised of 82 patients who underwent arthroscopic knee surgery over a one year period. Thirty patients were excluded from the study as they had additional ligamentous repair done or did not receive autologous conditioned plasma. The final study group comprised of 52 patients with a mean age of 44.56 ± SD12.74 years old. There were 16 females (31%) and 36 males (69%) in the study group. Twenty-five patients (48%) had left knee pain and 27 patients (52%) had right knee pain. Seventeen patients underwent concurrent meniscal repair. The mean time for presentation of symptoms was 19.84 ± 45.97 months. The mean follow-up post-operative until discharge from clinic was 6.50 ± 3.69 months.

Our study group had an average body mass index (BMI) of 28.89 ± 6.01 kg/m^2^. The average BMI for the group that failed, which has previously been described as worsening symptoms or need for further surgery, was lower at 25.4 ± 3.1 kg/m^2^ compared to 29.5 ± 6.2 kg/m^2^ for the group that did not fail.

The duration of surgery for the cases was 31.25 ± 9.43 minutes. In the group that performed well with surgery, the length of time of the operation was 31.5 ± 9.0 minutes compared to 30.0 ± 12.0 minutes for the failure group. The length of time off work as per medical certificate issued was 40.84 ± 27.08 days.

The baseline clinical characteristics of both patient groups are shown in Table I, which includes the BMI scores and severity of knee osteoarthritis (Kellgren-Lawrence Grading).

**Table I:** Characteristics of the study population

	Study population (n=52)	Improvement in symptoms (n=44)	Requiring further surgery/increase in pain (n=8)	p Value
Age (years)	44.56 ± 12.7	45.0 ± 13.2	42.4 ± 9.9	0.603
BMI (kg/m^2^)	28.90 ± 6.01	22.26 ± 2.88	22.84 ± 3.63	0.069
Sex			0.224	
Male	36 (69.2)	29	7	
Female	16 (30.8)	15	1	
Occupation				0.461
Deskbound	19	17	2	
Non-deskbound	33	27	6	
Operation time (minutes)	31.2 ± 9.44	31.5 ± 9.0	30.0 ± 12.0	0.688
Kellgren Lawrence scale (%)				0.43
0	0	0	0	
1	11 (21.1)	10	1	
2	7 (13.5)	5	2	
3	27 (51.9)	22	5	
4	7 (13.5)	7	0	
Dichotomized				
Kellgren-Lawrence scale (%)				0.85
Good 0-2	18 (34.6)	15	3	
Worse 3-4	34 (65.4)	29	5	
Medical leave (days)	40.8 ± 27.0			

In the study group, it was found that 11 patients were Grade 1 (21.2%), seven patients were Grade 2 (13.5%), 27 patients were Grade 3 (51.9%) and eight patients were Grade 4 (13.5%).

When comparing the pre-op range of motion of the knee and the post-operative range of motion of the knee on discharge, it was noted that 17 patients (32.7%) had improvement in their range while 32 patients (61.5%) had similar to preoperative range. However, three patients (5.8%) had worsening range of motion.

**Fig. 1:**
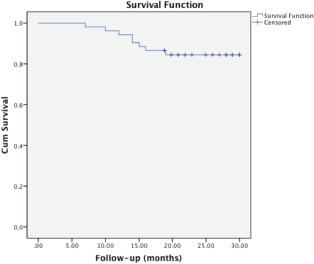
Kaplan-Meier Analysis.

In regards to their occupation, 19 patients (36.5%) had deskbound jobs while 33 patients (63.5%) were in physically active occupations. A Kaplan-Meier analysis was performed on the data gathered. It showed that the first failure would appear at seven months post index surgery. Subsequently, there would be further failures as the months progressed. At 18 months, the failure rate would stabilize and the graph plateaued. It can be assumed that patient who underwent arthroscopic debridement with autologous conditioned plasma would fail after seven months and thereafter would have their condition stabilized at around 18 months. The limitation to this analysis is that there might be more failures over a longer follow up period. (Fig. 1)

It was found that 44 patients (84.6%) had a favourable outcome following arthroscopic surgery with autologous conditioned plasma compared to eight patients (15.4%) who had unfavourable outcome. Of those eight patients, two patients had further surgery, and all still had pain despite the index surgery. In those with unfavourable outcomes, five patients had Kellgren-Lawrence Grade of 3, two patients had Grade 2 and one patient had a Grade 1. The t-test score had a p value of 0.43, thereby showing that the Kellgren-Lawrence grading indicating the extent of OA did not have a bearing on the outcome.

## Discussion

Autologous conditioned plasma is a concentrate extract of platelets from autologous blood. It represents a possible treatment option for stimulation and acceleration of soft tissue healing and regeneration. Concentrate extract of platelets are known to increase growth factor concentration three to five times that of normal plasma^9^. In the fields of sports medicine, it has been applied in the use of acute ligament injury, chronic tendon problems and around the knee joint. Most growth factors are secreted within one hour after intra-articular injections. Sanchez *et al* studied the effectiveness of plasma rich platelets (PRP) injection for OA of knees using hyaluronan injections for control group^10^.

Historically, arthroscopic debridement for OA knee has yielded successful outcome in 60%-80% of patient^11,12^. In our study, we have a success rate of 84.7%, slightly higher than that quoted in other studies. We can attribute this result to a younger population group that we treated as well as to careful patient selection and the latter complying with the rehabilitation protocol.

We looked at the different factors that we expected to have a bearing on the outcomes. We expected that body mass index and the severity of the osteoarthritis that the patient had would have had an adverse effect on the outcome. However, we did not find the correlation in our study. Arthroscopic debridement of symptomatic knee with autologous conditioned plasma seems to have beneficial outcome for patients. The majority of our patients had improvement in their symptoms at discharge on follow up clinic. In view of these findings, we recommend that arthroscopic debridement could be offered as a treatment option to patients who are symptomatic.

Careful patient selection criteria are as important as rehabilitation. Surgical options are not always the most appropriate as illustrated by the adage, “All surgeons know how to operate, a good surgeon knows when to operate, but a great surgeon knows when not to operate”^13^. Avoiding surgery may be appropriate when the level of pain appears disproportionate to the disorder and there is evidence of helplessness or failure to cope. Indeed, it is reported that between 15% and 30% of patients who undergo total joint replacement are dissatisfied with the outcome^14,15^. Mayr *et al *in a survey among experienced arthroscopic surgeons in Europe believed arthroscopy in osteoarthritis is appropriate under certain conditions^16^. The major task for surgeons is to select the right patients who are likely to benefit from this intervention. The respondent surgeons generally believed that an improvement is more likely in low-grade osteoarthritis and in neutral leg axis. The outcome was rated better if symptoms had persisted for less than six months and for patients that were younger than 60 years of age.

Limitations of our study would be the small number of patients and some patients underwent concurrent meniscal repair and the relatively short follow up period of no longer than one year post-operatively. The result of our paper might suggest that a randomized prospective study with patients undergoing arthroscopic debridement with and without autologous conditioned plasma will help to define the indications for the addition of autologous conditioned plasma more precisely.

## Conclusion

Based on the outcome analysis of our study, we advocate that arthroscopic knee debridement with autologous platelets injection should be considered as a treatment option in selected patients in whom joint arthroplasty is contraindicated or who are not keen to consider this option.
